# Do MRI findings identify patients with chronic low back pain and Modic changes who respond best to rest or exercise: a subgroup analysis of a randomised controlled trial

**DOI:** 10.1186/s12998-015-0071-x

**Published:** 2015-09-11

**Authors:** Rikke K. Jensen, Peter Kent, Mark Hancock

**Affiliations:** Research Department, Spine Centre of Southern Denmark, Hospital Lillebaelt, Institute of Regional Health Research, University of Southern Denmark, Oestre Hougvej 55, 5500 Middelfart, Denmark; Department of Sports Science and Clinical Biomechanics, University of Southern Denmark, Campusvej 55, 5230 Odense M, Denmark; Faculty of Human Sciences, Macquarie University, Balaclava Rd, North Ryde, 2113 NSW Australia

## Abstract

**Background:**

No previous clinical trials have investigated MRI findings as effect modifiers for conservative treatment of low back pain. This hypothesis-setting study investigated if MRI findings modified response to rest compared with exercise in patients with chronic low back pain and Modic changes.

**Methods:**

This study is a secondary analysis of a randomised controlled trial comparing rest with exercise. Patients were recruited from a specialised outpatient spine clinic and included in a clinical trial if they had chronic low back pain and an MRI showing Modic changes. All patients received conservative treatment while participating in the trial. Five baseline MRI findings were investigated as effect modifiers: Modic changes Type 1 (any size), large Modic changes (any type), large Modic changes Type 1, severe disc degeneration and large disc herniation. The outcome measure was change in low back pain intensity measured on a 0–10 point numerical rating scale at 14-month follow-up (*n* = 96). An interaction ≥ 1.0 point (0–10 scale) between treatment group and MRI findings in linear regression was considered clinically important.

**Results:**

The interactions for Modic Type 1, with large Modic changes or with large Modic changes Type 1 were all potentially important in size (−0.99 (95 % CI −3.28 to 1.29), −1.49 (−3.73 to 0.75), −1.49 (−3.57 to 0.58), respectively) but the direction of the effect was the opposite to what we had hypothesized—that people with these findings would benefit more from rest than from exercise. The interactions for severe disc degeneration (0.74 (−1.40 to 2.88)) and large disc herniation (−0.92 (3.15 to 1.31)) were less than the 1.0-point threshold for clinical importance. As expected, because of the lack of statistical power, no interaction term for any of the MRI findings was statistically significant.

**Conclusions:**

Three of the five MRI predictors showed potentially important effect modification, although the direction of the effect was surprising and confidence intervals were wide so very cautious interpretation is required. Further studies with adequate power are warranted to study these and additional MRI findings as potential effect modifiers for common interventions.

**Electronic supplementary material:**

The online version of this article (doi:10.1186/s12998-015-0071-x) contains supplementary material, which is available to authorized users.

## Background

In most patients with low back pain (LBP), the cause of pain cannot be definitively attributed to a specific pathology and patients are therefore labelled as having ‘non-specific LBP’. Non-specific LBP is estimated to be approximately 85 % of LBP in primary care [1]; however, most clinicians believe that it is not one condition but consists instead of several different subgroups. They also treat non-specific LBP differently depending on patterns of signs and symptoms [2] and preliminary results suggest that targeting treatment to LBP subgroups might be more effective than generic ‘one-size-fits all’ approaches [3].

There are many ways to potentially classify non-specific LBP into treatment-relevant subgroups, one of which is to use pathoanatomic findings seen on Magnetic Resonance Imaging (MRI). Although there is little evidence for the clinical relevance of most MRI findings, some, such as Modic changes, have been shown to be associated with LBP. A systematic review in 2008 [4] that investigated this relationship found positive associations between the presence of Modic changes and LBP in seven of 10 studies, with odds ratios ranging from 2 to 20. In addition, a stronger association with pain for Modic changes Type 1 than for other types was shown by Thompson et al. [5], who reported a higher positive predictive value for pain generation during discography for Modic changes Type 1 (0.81) than for Type 2 (0.64) or Type 3 (0.57).

Exercise therapy is a management strategy for non-specific LBP that is guideline-recommended and widely used [6]. As patients’ pathoanatomical source of pain is most likely diverse, it may be that MRI findings can identify subgroups of patients with chronic non-specific LBP who benefit more from exercise therapy than others. For example, on theoretical grounds, patients with chronic LBP and Modic changes could be a subgroup of patients that would be less likely to benefit from exercise, as the histology of Modic changes has shown fissured and disrupted endplates [7] that might indicate less tolerance of additional, exercise-induced, loading of the spine.

Based on the hypothesis that rest and reduction of spinal load would lead to better healing of the bone and subsequent reduction in pain, a two-group randomised controlled trial (RCT) investigated if rest was more effective than exercise [8] for people with Modic changes. The results showed no difference between the two treatment outcomes on pain, disability, quality of life or any other outcome measures immediately post-treatment (10 weeks) and at 14-month follow-up. A limitation of those results was that all patients had some type of Modic change, so it was not possible to determine if the presence of any Modic change acted as an effect modifier. However, we also collected information on the type and size of Modic changes as well as the presence or absence of other MRI findings such as disc herniation and disc degeneration. These data now provide the unique opportunity to investigate if the type or size of Modic changes, or the presence or absence of other MRI findings, acted as effect modifiers. To our knowledge, MRI findings have not previously been tested as effect modifiers for response to conservative interventions in an RCT, which seems a major gap in our knowledge.

Therefore, the purpose of this hypothesis-setting, secondary analysis was to investigate if MRI findings modified the treatment response to rest or exercise in patients with chronic LBP and Modic changes.

## Method

### Study design

This secondary analysis was performed using data from a (two-group) RCT that investigated the effect of rest compared with exercise in patients with chronic LBP and Modic changes. To increase the validity of this subgroup analysis, it was performed using the approach recommended by Sun et al. [9] which included pre-specification of both the direction of subgroup effects and the hypotheses underlying them, and the investigation of only a limited number of subgroups.

### Study population

Patients were recruited from a specialised outpatient spine clinic, the Spine Centre of Southern Denmark, where they had been referred by medical practitioners and chiropractors in primary care for investigation of non-response to conservative care. From August 2007 to December 2008, MRI was routinely performed on all patients meeting the following criteria: (i) no contraindications for MRI, (ii) LBP or leg pain of at least 3 on a 0–10 point Numerical Rating Scale (NRS), (iii) duration of current symptoms from 2 to 12 months, and (iv) age above 18 years.

Patients with an MRI showing Modic changes (Type 1, 2 or 3) that extended beyond the endplate into the vertebral body underwent a clinical examination and were invited to participate in the study unless they met any of the following exclusion criteria: (i) unable to participate in the project because of other physical or mental conditions, (ii) had symptoms and clinical signs of lumbar nerve root compression (e.g. leg pain dominating over back pain, positive straight-leg-raise test or neurological deficit), or (iii) had undergone previous spinal surgery with no pain relief after the operation.

### Randomisation and intervention

Patients were allocated to one of the two intervention groups (rest or exercise) by means of computerised minimisation software [10]. In total, 100 patients were included and of those, 49 were randomised to the rest group and 51 to the exercise group.

**Fig. 1:**
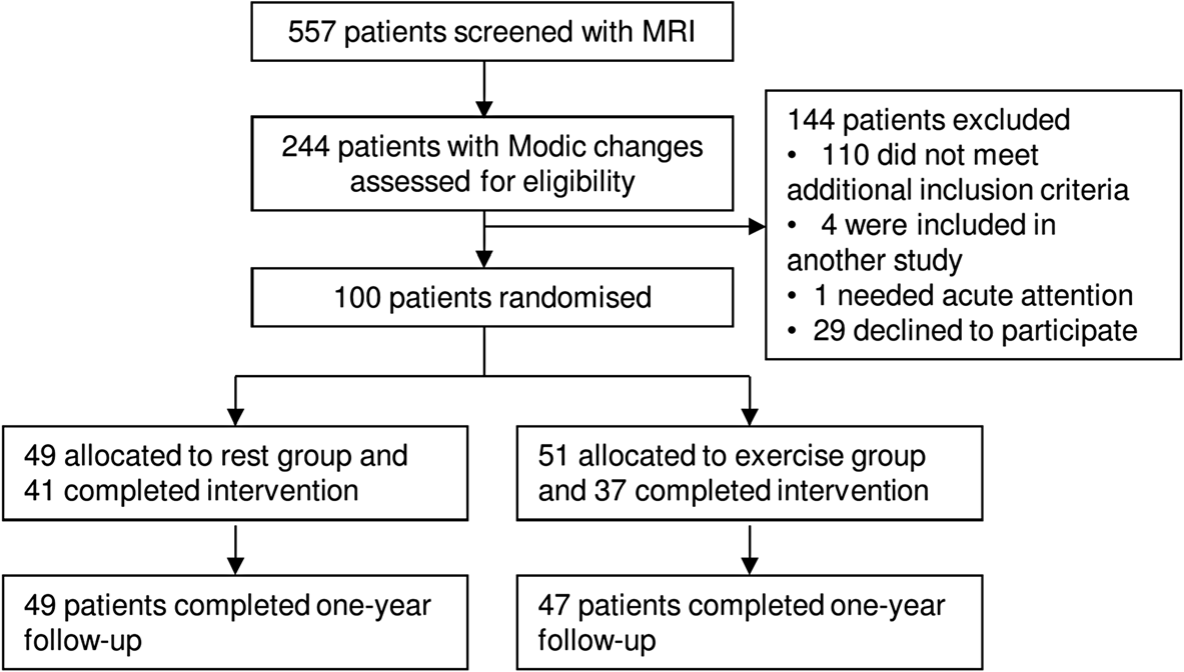
Flow of patients within the study

The rest group was instructed to avoid physically demanding activity and to rest twice daily for 1 h, by lying down. The patients participated in a group meeting once every second week to imitate the treatment session structure of the exercise group and thereby, the potential non-specific effect of being in a group. The exercise group received exercises for the stabilising muscles in the low back and abdomen together with dynamic exercises, exercises for postural instability and light physical fitness training. The patients exercised in a group once a week and were instructed to do additional home exercises three times a week. The duration of the interventions was 10 weeks and follow-up data were obtained at the end of this period (post-treatment at 10 weeks) and at 14 months after baseline. At 14 months, follow-up data on 96 participants were available (96 %). For the flow of the trial, see Fig. 1. Full details of the recruiting procedure and interventions have previously been reported [8].

### Outcomes

Current back pain measured on a 0–10 point NRS [11] was the primary outcome in the original trial, collected via self-reported questionnaire. For this secondary analysis, the change in current back pain between baseline and 14-month follow-up was chosen a priori as the treatment outcome, as this enabled the marginal means to be calculated for each subgroup.

### Variables of interest

MRIs were obtained at baseline. The MRI system was a 0.2 T (Magnetom Open Viva; Siemens AG, Erlangen, Germany) and a body spine surface coil was used with the patient in the supine position. The imaging protocol consisted of sagittal and axial T1- and T2-weighted sequences. The evaluation of the MRI changes (L1 to S1) was performed by an experienced musculoskeletal radiologist using standardised evaluation protocols [12, 13]. Previous evaluation of the use of these protocols by the same radiologist had shown substantial to almost perfect reproducibility with Kappa values from 0.73 to 1.0 for the Modic change variables [12] and from moderate to almost perfect with values from 0.59 to 0.97 for the disc-related changes [13]. The radiologist was blinded to any patient information except for name, age and sex.

Five potential effect modifiers for treatment with either rest or exercise were chosen from the baseline MRI variables and limited to that number to reduce the risk of type I error. The potential effect modifiers of interest were (i) type of Modic changes (Type 1 compared with not having Type 1 changes), (ii) size of Modic changes (large Modic changes compared with small), (iii) large Modic Type 1 changes (large Modic changes Type 1 compared with not having this finding), (iv) disc degeneration (severe disc degeneration compared with not having this finding) and (v) disc herniation (large disc herniations compared with not having this finding). The rationales for these variables are reported in Table 1.

**Table 1:** Rationale for variables

Modic changes Type 1 (compared with not having Type 1)
The histology of Type 1 shows fissured endplates and vascular granulation tissue adjacent to the endplate [7] and could potentially be an early state of bone healing. Therefore, we hypothesised that patients with Modic changes Type 1 would benefit more from rest than from exercise, as rest would facilitate bone healing compared with the compression forces added from exercise.
Large modic changes (compared with small ones)
Kuisma et al. [26] found that extensive Modic changes (≥25 % of vertebral height) were associated with a higher pain score in a working population. Large Modic changes could represent larger disruptions of the endplate and vertebral body and might therefore signal a better outcome from rest than from exercise.
Large modic changes Type 1 (compared with not having this finding)
Based on the hypotheses mentioned above for Modic type and size, we expected that people with large Modic changes Type 1 would benefit more from rest than from exercise.
Severe disc degeneration (compared with not having this finding)
Hancock et al. [27] reported that disc degeneration grade of ≥3 (Pfirrmann grade 1–5) was more than 5 times more likely to be present in patients with acute LBP than in controls without current LBP. Severe disc degeneration and mild disc degeneration could respond differently to conservative treatment. However, the available evidence does not clearly indicate a direction of a potential subgroup effect. Patients with severe disc degeneration could benefit from exercise due to the overall positive effects of physical activity. On the other hand, exercise could lead to increased load on a degenerated joint which could potentially result in a negative outcome.
Large disc herniation (compared with not having this finding)
Patients with LBP and sciatica receiving active conservative treatment [28] who also had broad-based protrusions and extrusions (‘large’ herniations) had a better outcome in leg pain and physical function than patients with disc bulges or focal protrusions. However, the evidence is sparse and it is possible that ‘large’ herniations could benefit either from the general effects of exercise or from less load with rest.

The definition of a patient being positive for *Modic changes Type 1* was the presence of a Type 1 finding on at least one of the 11 lumbar endplates and regardless of other types present on the same or other segmental levels. A patient was classified as being positive for *large Modic changes* if they had any type of Modic change, of ≥25 % of vertebral height on at least one of the 11 lumbar endplates regardless of other Modic changes present on other segmental levels. *Large Modic changes Type 1* was defined as having at least one Modic change which was both Type 1 and ≥25 % of vertebral height. In the MRI protocol [13], ‘disc height’ was graded from 0 to 3 and ‘disc signal intensity’ was graded from 0 to 3 (with higher numbers indicating more severe changes). For the purpose of this study, *severe disc degeneration* was defined as one or more discs with either (i) ‘disc height’ = grade 3, or (ii) the combination of ‘disc height’ = grade 2 and ‘disc signal intensity’ = grade 3 within the same disc. Disc herniations was evaluated according to the same MRI protocol [13], and for the purpose of this study, patients were classified as being positive for *large disc herniation* if they had one or more disc herniations categorised as broad-based protrusion, extrusion or a sequestration independent of the status of the other discs.

### Analysis

Data were analysed by linear regression models performed separately for each of the five potential effect modifiers. The dependent variable was change score in pain on a 0–10 point NRS (baseline score minus 14-month follow-up score). Each model included the treatment group variable, the potential effect modifier and the interaction term between the two. The interaction term was used to quantify size of the effect modification.

It has been estimated that the detection of a statistically significant subgroup interaction effect in an RCT requires a sample size approximately four times that required to detect a main effect of the same size [14]. Previous authors have suggested secondary analysis of RCTs as an approach to develop hypotheses for potentially important effect modifiers that can then be tested in suitably large trials [15]. As the current hypothesis-setting study was clearly underpowered, our focus was on the estimated effect size rather than statistical significance. If the interaction was greater than the threshold for MCID of 1.0 NRS points identified by Lauridsen et al. [16], we further explored the clinical interpretation by assessing the effect of intervention (rest compared with exercise) separately for those positive for the subgroup and negative for the subgroup, by calculating the marginal means for the subgroups. In addition, the number of patients achieving a MCID >1.0 point on a 0–10 NRS was calculated for those patients who were subgroup negative or positive.

### Ethics

This analysis was based on existing data collected for an RCT [8] approved by the Ethics Committee for the Region of Southern Denmark (approval # S-VF-20060111), registered in ClinicalTrials.gov (Identifier # NCT00454792) and performed following the Declaration of Helsinki principles. For all participants in the original RCT signed informed consent was obtained as required by the Ethics Committee for the Region of Southern Denmark. In Denmark, such secondary analysis does not require additional ethics approval (The Act on Processing of Personal Data, December 2012, Section 5.2; Act on Research Ethics Review of Health Research Projects, October 2013, Section 14.2).

## Results

Data from 49 patients in the rest group and 47 in the exercise group were available from the original RCT and were used for these analyses. The mean age was 46 years (range 21–60) and 69 % were women.

Participants in both treatment groups had similar socio-demographic and clinical characteristics at baseline, including age, sex, body mass index, type of occupation, sick leave, pain, activity limitation, general health, depression and expectations of treatment effect. Also, the distributions of the MRI variables of interest were similar between the two groups (Table 2). Distribution of the MRI variables per disc level is shown in Additional file 1.

**Table 2:** Distribution of MRI variables in the treatment groups

		Rest	Exercise	*p*-value
Modic changes Type 1	Yes	38	36	0.91
	No	11	11	
Large Modic changes (any type)	Yes	34	33	0.93
	No	15	14	
Large Modic changes Type 1	Yes	28	28	0.81
	No	21	19	
Severe disc degeneration	Yes	18	18	0.88
	No	31	29	
Large disc herniation	Yes	13	19	0.15
	No	36	28	

In the regression analyses, the interaction terms for type of Modic changes (Modic Type 1 compared with not having Type 1), size of Modic changes (large changes compared with small ones) and large Modic changes Type 1 (compared with not having this finding) were all greater than or approximated the 1.0-point threshold for clinical importance (Table 3). However, although we hypothesized that patients with these characteristics would benefit more from rest than from exercise, the direction of the effect was the opposite for all three variables. For example, the effect of rest versus exercise was less in participants with large Modic changes than in those with small Modic changes; the point estimate and 95 % CI for the interaction was −1.49 (−3.73 to 0.75). The interaction terms for disc degeneration and disc herniation did not meet the threshold for clinical importance (Table 3). As expected, none of the interaction terms for any of the MRI findings was statistically significant, most likely due to the small sample size.

**Table 3:** Results of linear regression models for change score in pain at 14-month follow-up

	Beta coefficient	*p*-value	95 % confidence interval
Modic changes Type 1			
Treatment^a^	0.82	0.42	−1.19;2.82
Modic changes Type 1	−1.79	0.03	−3.41; −0.17
*Interaction: Modic Type 1 & treatment*	*−0.99*	*0.39*	*−3.28;1.29*
Constant	2.09	0.004	0.67;3.51
Large Modic changes (any type)			
Large Modic changes	1.23	0.13	−0.37;2.84
Treatment	1.08	0.26	−0.80;2.95
* Interaction: large Modic changes & treatment*	*−1.49*	*0.19*	*−3.73;0.75*
Constant	−0.14	0.83	−1.49;1.20
Large Modic changes Type 1			
Large Modic changes Type 1	0.07	0.93	−1.42;1.55
Treatment	0.89	0.27	−0.70;2.47
*Interaction: large Modic changes Type 1 & treatment*	*−1.49*	*0.16*	*−3.57;0.58*
Constant	0.68	0.24	−0.46;1.83
Severe disc degeneration			
Severe disc degeneration	−0.002	0.998	−1.53;1.52
Treatment	−0.24	0.78	−1.55;1.07
*Interaction: severe disc degeneration & treatment*	*0.74*	*0.50*	*−1.40;2.88*
Constant	0.72	0.13	−0.22;1.67
Large disc herniation			
Large disc herniation	0.73	0.34	−0.78;2.24
Treatment	0.38	0.56	−0.90;1.66
*Interaction: large disc herniation & treatment*	*−0.92*	*0.42*	*−3.15;1.31*
Constant	0.43	0.38	−0.53;1.39

^a^Rest compared with exercise

As the interaction terms for type of Modic changes, size of Modic changes and large Modic changes Type 1 were larger than or approximated the 1.0-point threshold for clinical importance, therefore we further explored treatment effects for those in the subgroup compared with those not in the subgroup (Table 4). Patients with Modic changes Type 1 were 0.17 points (95 % CI −1.28 to 0.93) worse with rest than exercise, while those without Modic changes Type 1 were 0.82 points (−1.23 to 2.86) better with rest (Table 4). Patients with large Modic changes were 0.41 (−1.62 to 0.79) points worse with rest, while those without large Modic changes were 1.08 points better with rest (−0.97 to 3.12). Similar findings were identified for patients with large Modic changes Type 1 compared with those without this finding (Table 4). We also present the findings as the number of patients achieving an MCID in the subgroups in Table 5. As an example, of those with Modic changes Type 1, 7 % fewer patients reached the MCID if they received rest compared with exercise, while in those without Modic Type 1 changes, 9 % more reached the MCID if they received rest compared with exercise.

**Table 4:** Change in pain at 14-month follow-up in the two treatment groups

	Rest (mean (95% CI))	Exercise (mean (95% CI))	Treatment effect^a ^(mean (95% CI))
Modic changes Type 1	0.13 (−0.62;0.88)	0.31 (−0.47;1.08)	−0.17 (−1.28;0.93)
No Modic changes Type 1	2.91 (1.51;4.31)	2.09 (0.69;3.49)	0.82 (−1.23;2.86)
Modic changes large (≥25 %)	0.68 (−0.18;1.53)	1.09 (0.22;1.96)	−0.41 (−1.62;0.79)
Modic changes small (<25 %)	0.93 (−0.35;2.22)	−0.14 (−1.47;1.19)	1.08 (−0.97;3.12)
Large Modic changes Type 1 (≥25 %)	0.14 (−0.79;1.08)	0.75 (−0.18;1.68)	−0.61 (−1.82;0.61)
No large Modic changes Type 1	1.57 (0.49;2.65)	0.68 (−0.45;1.82)	0.89 (−0.93;2.70)

Change in pain (95 % CI) at 14-month follow-up on a 0–10 point NRS in the two treatment groups for type of Modic changes, size of Modic changes and large Modic changes Type 1

^a^Rest compared with exercise

**Table 5:** Number of patients achieving a Minimal Clinical Important Difference

Subgroup	Rest	Exercise	Treatment effect
Modic changes Type 1	24 % (*n* = 9)	31 % (*n* = 11)	7 % fewer patients in the rest group achieved MCID
No Modic changes Type 1	64 % (*n* = 7)	55 % (*n* = 6)	9 % more patients in the rest group achieved MCID
Modic changes large (≥25 %)	32 % (*n* = 11)	39 % (*n* = 13)	7 % fewer patients in the rest group achieved MCID
Modic changes small (<25 %)	33 % (*n* = 5)	29 % (*n* = 4)	4 % more patients in the rest group achieved MCID
Large Modic changes Type 1 ((≥25 %)	25 % (*n* = 7)	36 % (*n* = 10)	11 % fewer patients in the rest group achieved MCID
No large Modic changes Type 1	43 % (*n* = 9)	37 % (*n* = 7)	6 % more patients in the rest group achieved MCID

Number of patients (in percentage) achieving a Minimal Clinical Important Difference (MCID >1.0-point on a 0–10 point NRS) at 14-month follow-up in the two subgroups

**Fig. 2:**
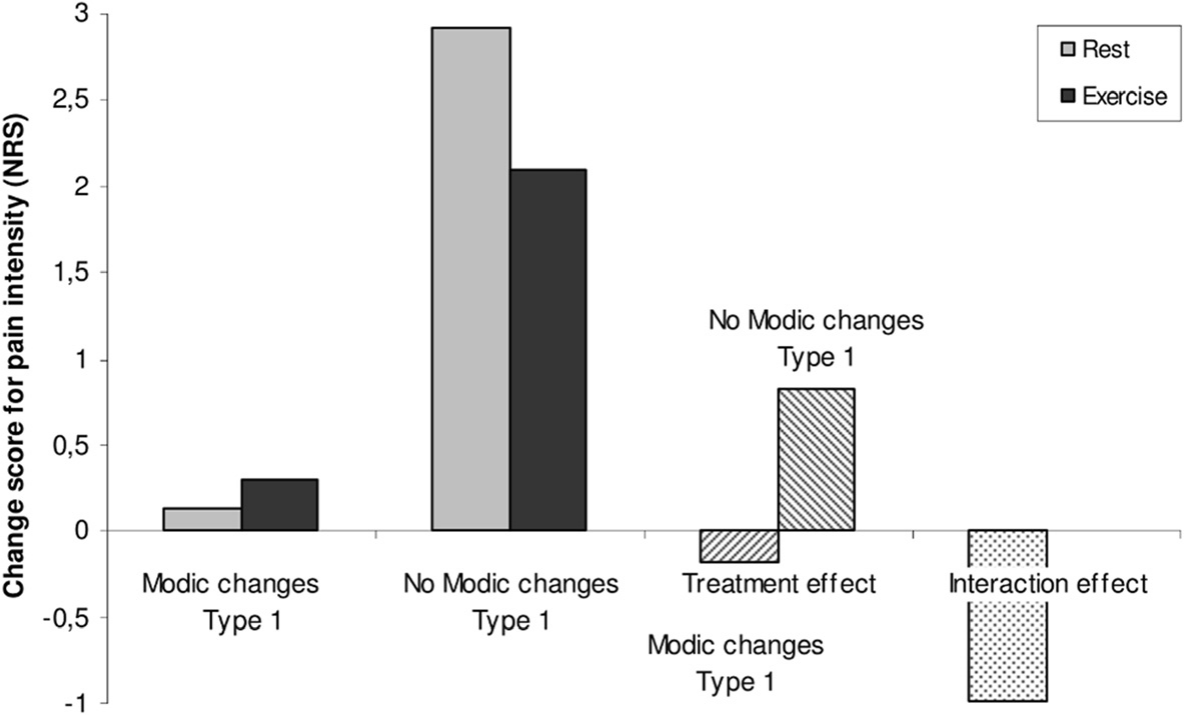
Treatment effect in patients with and without Modic changes Type 1. Comparison of changes in pain and the treatment effect (rest compared with exercise) in patients receiving rest or exercise subgrouped into those with and without Modic changes Type 1

**Fig. 3:**
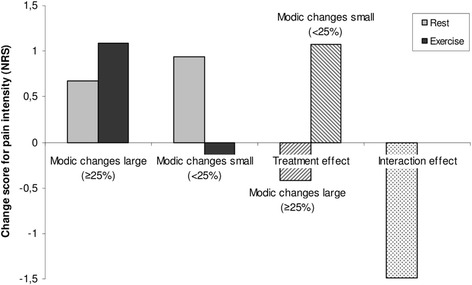
Treatment effect in patients with and without large Modic changes. Comparison of changes in pain and the treatment effect (rest compared with exercise) in patients receiving rest or exercise subgrouped into those with and without large Modic changes

**Fig. 4:**
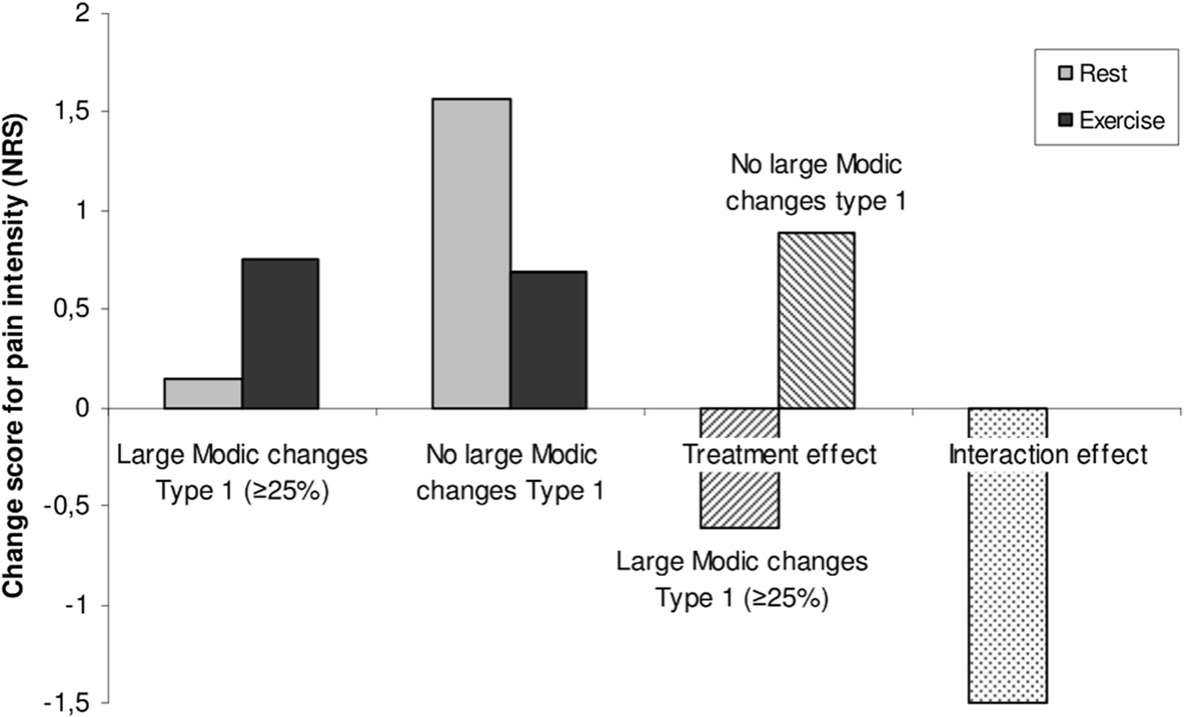
Treatment effect in patients with and without large Modic changes Type 1. Comparison of changes in pain and the treatment effect (rest compared with exercise) in patients receiving rest or exercise subgrouped into those with and without large Modic changes Type 1

A graphical display of the comparison of outcome in pain for the two treatment groups for the three potential effect modifiers reaching the threshold of 1.0 point, together with treatment effect for those in the subgroup or not in the subgroup (i.e. MRI finding positive vs. MRI finding negative) and interaction effect is shown in Figs. 2, 3 and 4.

## Discussion

### Statement of principal findings

To our knowledge, this is the first study investigating MRI findings as effect modifiers for response to conservative interventions in a LBP RCT. Although none of the interaction terms for any of the MRI findings tested were statistically significant, Modic changes Type 1, large Modic changes and large Modic changes Type 1 showed tentative evidence of effect modification that was potentially important in size (point estimates ranging from −0.99 to −1.49 on the NRS). Surprisingly, the direction of the effect modification was opposite to those specified in our hypothesis and this is further reason for caution when interpreting the findings. That all three results were consistently in the direction opposite to our hypothesis suggests the notion, that exercise would aggravate physically larger or early stage Modic changes, may have been biologically plausible but overly simplistic. Although this study was underpowered, thereby increasing the risk of the results being due to chance, it suggests which of these potential effect modifiers might be analysed in subsequent studies and provides data suitable for calculating sample sizes for such studies.

### Strengths and weaknesses of the study

A strength of the current study is that it was based on data from an RCT, which is the definitive type of data in which to quantify effect modification [17]. In addition, the MRIs were reported using a standardised protocol by a radiologist who had previously demonstrated consistency in evaluating spinal pathologies and was blinded to both the treatment group and the clinical outcome, however, in other settings and with other MRI variables the reliability could vary. Our choice of MRI variables as potential effect modifiers was not exhaustive and other variables could have been potentially important. However, as recommended by Sun et al. [9], we limited the number of variables to cautiously selected potential effect modifiers with, whenever possible, pre-specified assumptions about the direction of the effect. The hypotheses were built on the literature but, as this was often sparse, the likely direction of the effect was not always obvious a priori and we took the pragmatic view that an effect modifier might have potential importance regardless of the direction of the effect. A limitation to the generalisability of the results is that patients in the study population all had some type of Modic changes.

In a study by Bendix et al. [18] investigating Modic changes using low-field MRI (0.3 T) compared with high-field MRI (1.5 T), the authors found a difference in the prevalence rate, with Modic changes Type 1 being detected three times more often using low field MRI, whereas Type 2 was detected two times more often when using high field MRI. As the MRI system used in the current study was low-field (0.2 T), this may have affected the observed prevalence of Type 1 and 2, but it is unknown whether one MRI approach is more accurate than the other or simply more sensitive and less specific. In addition, using only T1- and T2-weighted sequences to identify the type of Modic change may add to the uncertainty of identifying Modic changes Type 1, as this is optimally visualised using a fluid sensitive (STIR) sequence which was not used in the current study.

The MCID was set to 1 point, which is a small difference that is at the lower end of reported estimates for MCIDs. However, the MCID we used was based on the value estimated from a previous chronic LBP sample from the same hospital department and therefore is likely to be the most appropriate for our study sample [16]. Similarly, the RCT from which the data is used in the current study showed that these patients’ pain scores, on average, changed very little over 1 year (0.8 (95 % CI 0.3 to 1.3)) suggesting that their MCID would also likely be low [8].

### Meaning of the study and comparison with other studies

To our knowledge, there have been very few RCTs that have formally investigated treatment effect modifiers for exercise therapy [19] and previous attempts to identify subgroups of responders to exercise have used aspects of the clinical presentation. For example, Long et al. [20] found that short-term activity and short-term pain limitation were improved in people with a directional preference, if that exercise was matched to their directional preference rather than being unrelated to that preference. In the current study, we have taken a different approach by focusing on MRI findings rather than aspects of the clinical presentation. Both approaches may yield useful evidence but clearly there is a need to improve the effects of exercise by better targeting [19].

There have also been few previous studies of MRI findings as effect modifiers for LBP or sciatica treatments and, to our knowledge, all have investigated invasive interventions, such as injections and various types of surgery. Two of these studies found evidence of significant treatment effect modification (calculations based on data presented in manuscripts) [21, 22]. One study found that people with LBP and Modic changes Type 1 (compared with Modic changes Type 2) had less activity limitation following Disprosan (steroid) injections than if they had saline injections [21]. The other found that sciatica patients with central disc herniation (compared with those without central disc herniation) had less pain following surgery than if they had rehabilitation instead [22]. As early activation and exercise are the most widely recommended treatment for LBP [6], it seems an oversight to not investigate pathoanatomic findings as potential effect modifiers for response to exercise.

Investigation into MRI findings as effect modifiers is complex for a number of reasons. A spinal MRI contains a large amount of anatomical information that requires a detailed protocol to be comprehensively described. When working with a dataset of small sample size, testing for effect modification inevitably leads to data reduction being required and this risks overlooking potentially important information such as location of disc herniation, signal intensity in herniation, location of Modic changes or irregular endplates. Also, numerous MRI findings are present at the same time at a segmental vertebral level and also across all five lumbar segments. For example, vertebral endplate signal changes (Modic changes) and vertebral disc herniation almost always co-exist with other degenerative disc findings, such as reduction of height and signal intensity of the disc [23, 24]. One approach to integrating this multiplicity would be to adjust for the co-existence of other MRI findings in the statistical modelling, but in this study that was not possible due to the lack of power. Alternatively, other statistical methods such as Latent Class Analyses could be used to define clusters of MRI findings and those clusters could then be tested as potential effect modifiers, instead of solitary MRI findings [25]. However, those types of analyses using MRI findings are still very novel and require further validation.

## Conclusion

In this study, Modic changes Type 1, large Modic changes and large Modic changes Type 1 showed potentially important treatment modification effects by meeting or exceeding our threshold of a 1.0-point difference in pain intensity (0–10 NRS) at 14-month follow-up. Severe disc degeneration and large disc herniations did not reach that threshold. The results need to be interpreted very cautiously as this was a hypothesis-setting study with a relatively small sample, none of the potential effect modifications reached statistical significance, and the effects were in the direction opposite to our hypothesis. Despite this, the findings can be used to indicate some MRI effect modifiers suitable for investigation in subsequent studies of LBP treatment effect and these estimates of effect could be used to adequately power those studies.

## Additional file

Additional file 1:
**Distribution of MRI variables per disc level.** Distribution of type and size of Modic changes per disc level (one disc level = 2 endplates) in 96 patients (1056 endplates) with low back pain and Modic changes. Table B. Distribution of severe disc degeneration and type of herniation per disc level in 96 patients (480 discs) with low back pain and Modic changes. (PDF 35 kb)

## Abbreviations

LBP: Low back pain
MRI: Magnetic resonance imaging
RCT: Randomised controlled trial
NRS: Numerical rating scale
MCID: Minimal clinically important difference
CI: Confidence interval
STIR: Short tau inversion recovery
