# Supplementary material for: Do MRI findings identify patients with chronic low back pain and Modic changes who respond best to rest or exercise: a subgroup analysis of a randomised controlled trial
Source: Chiropr Man Therap. 2015 Sep 11;23:26. doi: 10.1186/s12998-015-0071-x (PMC4566202; doi:10.1186/s12998-015-0071-x)
Supplement: Additional file 1: — Distribution of MRI variables per disc level. Distribution of type and size of Modic changes per disc level (one disc level = 2 endplates) in 96 patients (1056 endplates) with low back pain and Modic changes. Table B. Distribution of severe disc degeneration and type of herniation per disc level in 96 patients (480 discs) with low back pain and Modic changes. (PDF 35 kb) [file 12998_2015_71_MOESM1_ESM.pdf]

Additional file 1

Table A
Distribution of type and size of Modic changes per disc level (one disc level = 2 endplates) in 96 patients (1056 endplates) with low back pain and Modic changes.

		Type of Modic changes (n)						Size of Modic changes (n)			
**Disc level**	**No MC**	**Type I**	**Type II**	**Type III**	**Mixed I/II**	**Mixed II/III**	**Mixed I/III**	**EP only**	**<25%**	**25-50%**	**>50%**
**Th12/L1***	95	1	0	0	0	0	0	1	0	0	0
**L1/L2**	182	2	4	0	2	0	2	1	6	0	3
**L2/L3**	172	8	10	0	0	0	2	5	6	4	5
**L3/L4**	160	14	11	0	3	2	2	7	16	5	4
**L4/L5**	126	23	36	0	7	0	0	17	18	18	13
**L5/S1**	59	57	31	0	29	12	4	14	49	48	22
**Total**	**794**	**105**	**92**	**0**	**41**	**14**	**10**	**45**	**95**	**75**	**47**

***In this disc level only the upper endplate of L1 is evaluated. EP=endplate.**
The 'EP only' category is artificial small as patients were only included in the original trial if they had at least one level with Modic changes extended beyond the endplate into the vertebral body.

Table B
Distribution of severe disc degeneration and type of herniation per disc level in 96 patients (480 discs) with low back pain and Modic changes.

	Degeneration (n)	Type of herniation (n)					
**Disc level**	**Severe disc degeneration**	**Normal**	**Bulging**	**Focal**	**Broad-based**	**Extrusion**	**Sequestration**
**L1/L2**	4	75	20	1	0	0	0
**L2/L3**	4	63	31	1	0	1	0
**L3/L4**	6	50	41	3	2	0	0
**L4/L5**	13	21	50	13	6	6	0
**L5/S1**	24	20	43	14	5	13	1
**Total**	**51**	**229**	**185**	**32**	**13**	**20**	**1**
